# Ectoine protects DNA from damage by ionizing radiation

**DOI:** 10.1038/s41598-017-15512-4

**Published:** 2017-11-10

**Authors:** M.- A. Schröter, S. Meyer, M. B. Hahn, T. Solomun, H. Sturm, H. J. Kunte

**Affiliations:** 1 Federal Institute for Materials Research and Testing, D-12205 Berlin, Germany; 2 Institute of Biochemistry and Biology, University of Potsdam, D-14476 Potsdam, Germany; 3 Institute of Experimental Physics, Free University Berlin, Department of Physics, D-14195 Berlin, Germany; 4 Technical University Berlin, D-10587 Berlin, Germany

## Abstract

Ectoine plays an important role in protecting biomolecules and entire cells against environmental stressors such as salinity, freezing, drying and high temperatures. Recent studies revealed that ectoine also provides effective protection for human skin cells from damage caused by UV-A radiation. These protective properties make ectoine a valuable compound and it is applied as an active ingredient in numerous pharmaceutical devices and cosmetics. Interestingly, the underlying mechanism resulting in protecting cells from radiation is not yet fully understood. Here we present a study on ectoine and its protective influence on DNA during electron irradiation. Applying gel electrophoresis and atomic force microscopy, we demonstrate for the first time that ectoine prevents DNA strand breaks caused by ionizing electron radiation. The results presented here point to future applications of ectoine for instance in cancer radiation therapy.

## Introduction

Ectoine (1,4,5,6-tetrahydro-2-methyl-4-pyrimidinecarboxylic acid) is synthesized and accumulated in molar concentration by bacteria to withstand osmotic stress^1–3^. Even at high molar concentration, ectoine and related substances do not disturb the metabolic pathways within the cell and are therefore called compatible solutes^4^. Ectoine functions not only as an osmoregulatory compatible solute but also protects cell components and even whole cells against different stressors such as freezing and thawing, high temperatures, and drying^1,5–7^. In addition, it was shown that ectoine mitigates the damages on eukaryotic cells caused by ultraviolet radiation. The study by Bünger and coworkers revealed a decrease in mutations in mitochondrial DNA of human skin cells when these cells were incubated with ectoine prior to irradiation with UV-A light^8^. Similar results were obtained after irradiation with UV/VIS photons and less damage to DNA was observed in cells that have been treated with ectoine.

The mechanism by which ectoine protects cell components such as proteins and membranes is relatively well understood^5^. The beneficial effect is explained by the lower affinity of ectoine, compared to water, to the surface of such biomolecules. The low affinity results in a thermodynamic force that contributes to increased stability of proteins and membranes^9^. The mechanism in protecting living cells against UV radiation, however, is far from being clear. Ectoine reduces the release of certain inflammatory factors and thus it was postulated that ectoine diminishes inflammatory processes caused by UV radiation^10^. Likewise, it is hypothesized that ectoine-induced expression of heat shock proteins is the cause for protection against UV radiation^11^.

In the present study it was investigated whether ectoine is able to protect DNA from damage by ionizing electron radiation in a cell-free setting. Plasmid DNA was irradiated with high energy electrons (30 keV) in water containing 1 M ectoine. Applying gel electrophoresis and atomic force microscopy (AFM) it was found that ectoine on its own is a potent protective substance of DNA against ionizing radiation.

## Results

To find out whether ectoine protects DNA from radiation, plasmid pUC19 (2686 bp) was dissolved in ultrapure water (pH 6.6) or water containing ectoine (1 M) and exposed to electron beams of 30 keV with varying currents of 0 to 12.7 nA. The primary electrons were generated with the aid of an electron microscope and the experimental setup is described in detail by Hahn *et al*.^12^.

Plasmid DNA has been used for the experiments because its supercoiled isoform is highly sensitive to radiation damage. Assuming one nucleotide unit measures 0.34 nm^13^, the entire contour length of pUC19 plasmid is nearly 913 nm. Intact plasmid DNA appears as supercoiled (sc) structure. Single strand breaks (ssb) due to mechanical stress or enzymatic activity will lead to an open circular (oc) isoform, whereas double strand breaks (dsb) will result in linearized (lin) DNA. Further degradation of linearized DNA will result in even shorter fragments.

**Figure 1:**
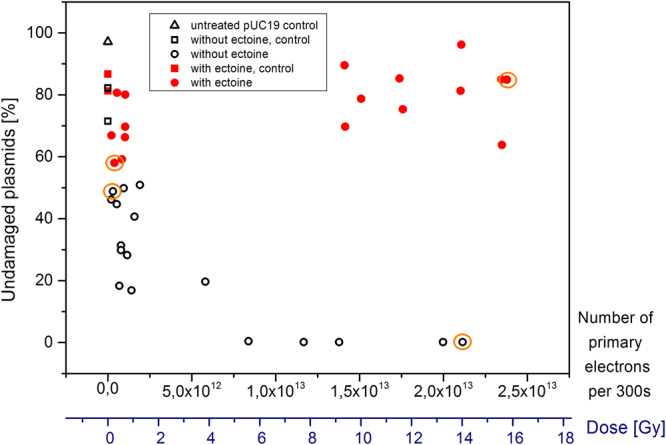
Undamaged plasmids per pUC19 samples irradiated with electrons (30 keV) in pure water (pH 6.6) and water containing 1 M ectoine.

Applying gel electrophoresis and intermittent contact AFM enabled identification and quantification of different plasmid isoforms. After electrophoretic separation, GelRed-stained DNA isoforms were quantified from agarose gels and the amount of undamaged plasmids was plotted against the number of primary electrons and against the dose, respectively (Fig. 1). The different isoforms were assigned according to their position in the gel by comparison with native supercoiled pUC19 DNA and its linearized isoform (*Hin*dIII digest). Furthermore, binding efficiencies of GelRed for the same amount (75 ng) of sc and linear isoform DNA were determined and the correction factor was obtained as 0.95 ± 0.01. The minimally weaker binding to scDNA could therefore be neglected.

To estimate how much the sample treatment prior to irradiation has already changed the structure of pUC19, two different control samples were compared. The first control was completely untreated plasmid DNA, which consisted of approximately 97% supercoiled DNA. Plasmid DNA that went through the entire preparation process until irradiation was the second control and contained in average of 77% scDNA (ultrapure water, two replicates) and 84% scDNA (with ectoine, two replicates), respectively.

Plasmid pUC19 DNA was irradiated for 300 s in water and by increasing the number of primary electrons, we observed a strong decline in undamaged scDNA until the complete loss of scDNA. In contrast, irradiation of pUC19 with high amount of primary electrons in the presence of 1 M ectoine, kept most of the DNA intact. On average, 81% of the plasmids remained in the native supercoiled isoform, however, with a standard deviation of approximately 9%.

Quantification of GelRed-stained DNA via relative fluorescence signals from agarose gels. The black triangle shows a control sample without any treatment. Black circles show samples in water (no ectoine) and red circles represent samples in 1 M ectoine solution. The data denoted by black and red squares are obtained from samples that went through the entire preparation process, which includes the incorporation in the microscope chamber, but without any radiation treatment. Additionally, representative samples, highlighted with a yellow circle, are analysed with AFM to obtain the distribution of the pUC19 isoforms (Table 1).

**Table 1:** Quantitative results at selected data points (compare Fig. 1) from gel electrophoresis and AFM outlining the formation of different pUC19 isoforms during irradiation with electrons, performed with and without ectoine.

pUC19	number of primary electrons [**∙**10¹²] per 300 s	Effective irradiation dose [Gy]	undamaged plasmids		damaged plasmids				
			supercoiled		open circular		linearized (~913 nm)		fragmented
			Gel data Relative Fluorescence signal %	AFM data Mean % ± STD	Gel data Relative Fluorescence signal %	AFM data Mean % ± STD	Gel data Relative Fluorescence signal %	AFM data Mean % ± STD	AFM Data
without ectoine	0.305	0.2	49	63 ± 15	51	35 ± 13	0	2 ± 3	no
	21.14	14.07	*	**	*	**	*	**	yes**
with ectoine (1 mol/l)	0.394	0.26	58	60 ± 3	42	33 ± 3	0	7 ± 2	no
	23.78	15.83	85	84 ± 8	15	15 ± 7	0	1 ± 1	no

*Only ocDNA and linear DNA, respectively, were detected. Quantification was not possible since ocDNA and linear DNA could not be distinguished by gel electrophoresis.

**Highly fragmented DNA was the main DNA species found by AFM.

For further details see discussion.

The fraction of undamaged plasmids after irradiation with different electron doses was quantitatively also analyzed by means of intermittent contact AFM, which has developed into a standard technique for imaging susceptible biomolecules^14^ with nanometer resolution. In particular, AFM has been used for structural analysis of supercoiled, open circular and linearized plasmid DNA as well as for analysis of contour lengths of linear DNA, which was fragmented by radiation^15–18^.

Hence, within the seventeen different irradiated samples we selected four different and characteristic representatives. The chosen samples comprised pUC19 DNA dissolved in unbuffered water, which was irradiated with a low and a high electron dose of 0.2 Gy and 14.07 Gy, respectively, and pUC19 dissolved in 1 M ectoine solution, which was irradiated with 0.26 Gy and 15.83 Gy, respectively (see supplementary information, Table S1).

**Figure 2:**
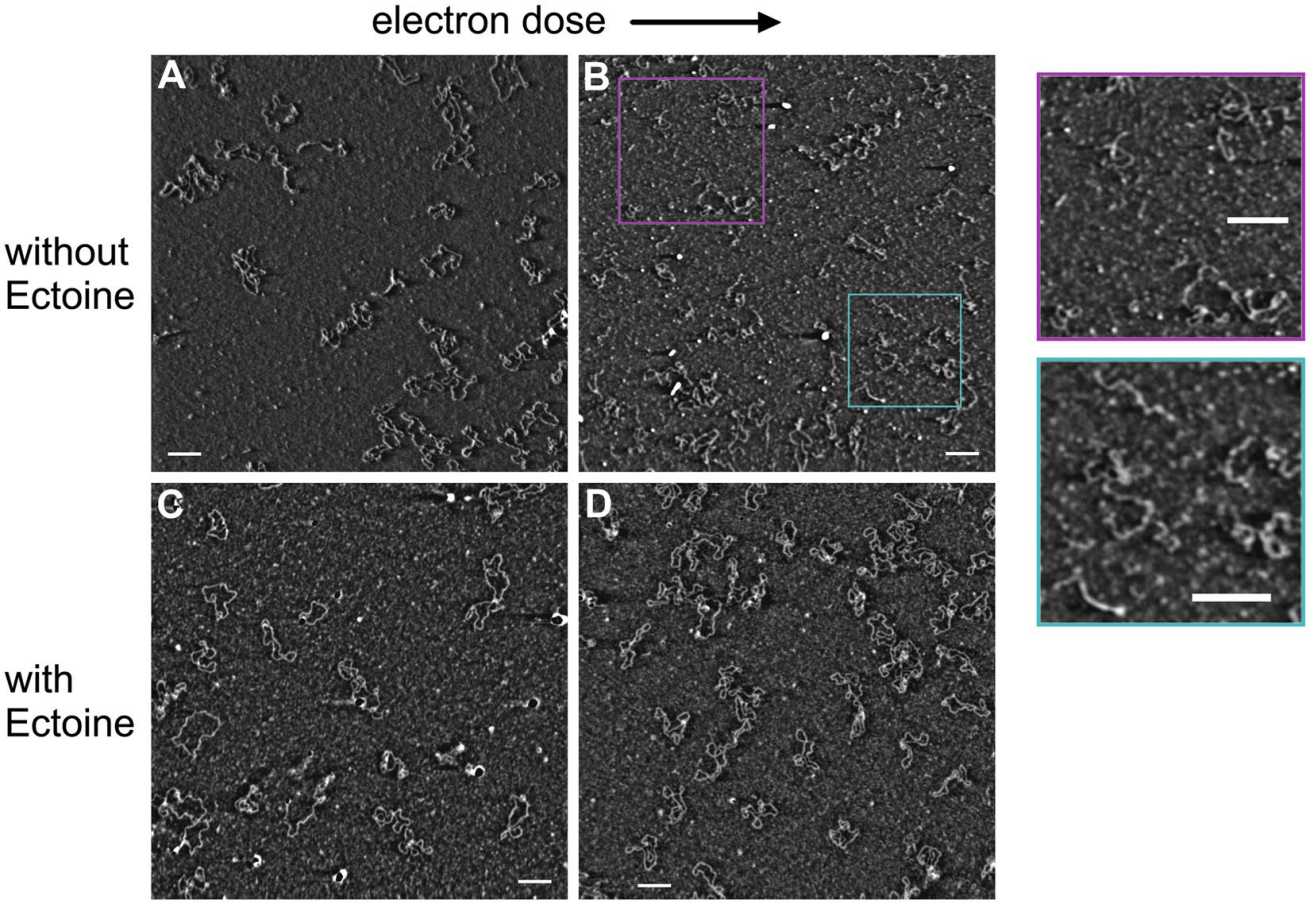
Representative AFM amplitude images of electron-irradiated pUC19 DNA. Plasmid pUC19 in water was irradiated with a low electron dose of 0.2 Gy (**A**) and with a high electron dose 14.07 Gy (**B**). With increasing dose the scDNA declines and linear DNA arises that varies greatly in length (**B** and display details thereof). In comparison, pUC19 in 1 M aqueous ectoine solution after irradiation with 0.26 Gy (**C**) and 15.83 Gy (**D**) stays in its undamaged and native scDNA isoform. For AFM imaging the DNA was chemically fixed on ultra-smooth mica as a substrate. (bar = 200 nm).

For statistical reasons, three replicates were prepared from each of the four solutions on mica substrate. From each mica surface at least three different sites were picked and multiple images were scanned from these sites by atomic force microscopy. Four representative AFM images of electron irradiated pUC19 DNA are shown in Fig. 2. The amount of DNA isoforms that was found in the water samples by AFM imaging was analyzed, means and standard deviation of the three replicates are given in Table 1. With increasing radiation dose the number of scDNA declines and fragmented DNA arises. These results are in good agreement with the data obtained by gel electrophoresis, which revealed the complete loss of scDNA after high-dose irradiation (compare Fig. 1) and an increase in the formation of open circular and linear DNA, respectively (Table 1). Although linear DNA migrates somewhat faster compared to the open circular isoform, a reliable discrimination of both forms by electrophoresis was often difficult. In contrast, AFM allowed to distinguish all three DNA isoforms based on single molecule analysis (Fig. 2) and even small amounts of linear DNA could be seen in samples irradiated with 0.2 Gy. Applying a radiation dose of 14.07 Gy, AFM analysis revealed an increase in linear DNA of varying sizes, while scDNA, ocDNA, and full length linear DNA (~913 nm) were detectable only in low quantities.

Dissolving pUC19 DNA in aqueous ectoine solution (1 M) changes the effect of radiation on DNA and ectoine apparently confers protection even against high radiation of 15.83 Gy. In the presence of the compatible solute, the plasmid remained predominantly in the supercoiled isoform irrespective of the applied radiation dose (compare Figs 1 and 2C and D).

## Discussion

Analyzing DNA by gel electrophoresis and AFM showed that ionization irradiation (30 keV electrons) causes strand breaks in supercoiled plasmid DNA. The present study demonstrates for the first time that ectoine prevents DNA from being damaged by ionizing electron radiation. The argumentation behind the statement above is based on the following approach.

Strand breaks become apparent through changes in DNA conformation, which leads to the appearance of open circular and linear DNA isoforms and the decline of supercoiled DNA. Electrophoresis is a well-established method for structural analysis of plasmids. Because irradiated plasmids undergo conformational changes, altered electrophoretic mobility can be exploited for quantification, even though short fragments below 200 bp are difficult to detect^17,19,20^. Atomic force microscopy allows to visualize changes in DNA conformation. The number of single- and double-strand breaks introduced into a single DNA molecule represents the level of damage. Hence, the length distribution of the fragments is of great interest for quantifying the damage caused by radiation.

Ionizing radiation is known to disintegrate DNA. The large number of short fragments is the result of densely localized ionization events in the proximity of the DNA. Hence, it is widely accepted that this is leading to destabilising the secondary structure of DNA^14–18^. Depending on the dose and the type of radiation, the effect of DNA lesions are ranging from cross-links, base releases and combination of single strand-breaks, double strand-breaks to clustered double-strand breaks^21–23^.

The damaging effects on DNA dissolved in water have been found to be three magnitudes of order higher compared to radiation experiments with dry DNA^24–26^. The mechanisms by which the damage to DNA in water is intensified was explained by the formation of secondary particles such as OH-radicals, H-radicals, ions, prehydrated electrons and low energy electrons. These particles are generated largely by interactions of ionizing radiation with water molecules^27^. The most abundant and damaging species are secondary electrons and radicals. The majority of secondary electrons under the given irradiation conditions have energies below 100 eV, which is sufficient to damage biomolecules directly or produce reactive radicals by ionization of water as was determined by Monte-Carlo simulations in our previous studies^28,29^.

The here described effects of ionizing radiation (30 keV electrons) on DNA dissolved in water are also in good agreement with results from our previous studies^27^. Additionally, the amount of different plasmid isoforms that have been detected by gel electrophoresis and AFM are in good agreement (compare Table 1). However, after irradiating DNA with 14.07 Gy in pure water, the results from gel electrophoresis and AFM differ in some respects. Even though silanization of mica formed a granular substructure hindering a quantitative analysis of fragments <100 bp an increase of small particles was observed and attributed to small fragments (Fig. 2B).

Applying gel electrophoresis, ocDNA and linear DNA were found, whereas almost completely fragmented DNA, as was to be expected^16,18,20^, was detected predominantly by AFM imaging. Comparing both detection principles it is obvious, that results from gel electrophoresis as an integrating method have their limits concerning the detection of small molecules. On the contrary, AFM suffers from a limited scan field but benefits from high spatial resolution needed to detect highly damaged species. Such inconsistencies between AFM and electrophoresis were also found by Jiang *et al*.^19^.

Some authors have proposed that such differences may result from the chemical fixation on mica during AFM sample preparation, which introduces further strand breaks into already damaged DNA and thereby generating short linear molecules^19^. It is known that adhesion of DNA to mica surfaces with an incomplete silane layer can change the molecule’s conformation^30^. As described by Schmatko and coworkers^30^, the charges on a bare surface without silane can cause damage to DNA and transform supercoiled pUC19 into open circular DNA. However, such conformational changes of control samples into linearized or even fragmented DNA were not observed by AFM in our experiments. During sample preparation, each mica surface was visually assessed to ensure that the surface was completely silanized. Thus, it was made sure that the observed damage to DNA was caused by radiation and not by sample preparation or substrate adhesion. For gel electrophoresis, it is known that due to molecular entanglement DNA molecules could be transported in the “wrong” gel band^19^ and are indistinguishable from each other in terms of their conformation. In support of our findings, Murakami and coworkers^17^ reported that after ^60^Co exposure short fragments of DNA can be clearly seen by AFM, whereas those fragments escaped detection by gel electrophoresis. The experiments carried out here, clearly revealed that the compatible solute ectoine protects DNA from damage caused by electron radiation and preserves its native supercoiled conformation, even if inconsistencies between the methods are still worth to be discussed.

Descriptions of the mechanisms by which ectoine protects DNA against ionizing electron radiation are still under discussion. Ectoine and other compatible solutes protect proteins from denaturation by stressors such as heat or dryness^5,9^. Stabilizing the native folded state is explained by the preferential exclusion of compatible solutes from the surface of proteins and their first hydration shell. Stabilization by preferential exclusion is explained as the result of the tendency to minimize the surface area^9,31^. However, this model might not hold true for all molecules. Based on atomistic molecular-dynamics simulations for the compatible solute hydroxyectoine, it was shown that hydroxyectoine can be attracted to surfaces of *negatively* charged molecules.

Following the preferential exclusion model, the attraction of hydroxyectoine to negative surfaces was coined preferential binding^32^. Firstly, the model based on the molecular dynamics simulation confirmed the preferential exclusion of hydroxyectoine from the surface of *positively* charged proteins and their first hydration shell^33–35^. Secondly, the model revealed a preferential binding of hydroxyectoine to *negatively* charged spheres such as DNA within a distance of 0.6 nm above the surface^32^. As a consequence, hydroxyectoine will partially replace the first water shell that surrounds DNA in a distance of approximately 0.2 to 0.5 nm^36–38^. Assuming that ectoine resembles hydroxyectoine in terms of its binding properties, it is suggested that ectoine as well partially replaces water from the hydration shell of DNA.

Thus, the mechanism by which ectoine stabilizes DNA against ionizing electron radiation can be described as follows: For the interaction of ionizing radiation with water we can assume that a decrease of degradative species in the vicinity of DNA takes place and thus mitigates the damage of DNA. Additional effects such as the scavenging of radicals are already under investigation.

In contrast to other studies, the calculation of the radiation doses in this survey is based on a microdosimetric approach^27^. In our previous study the energy deposit per volume of plasmid including its first hydration shell and per primary electron (30 keV) in water through a 100 nm thick Si_3_N_4_ membrane has been found to be 1.205∙10^−14^ eV^27^. Although in the experimental setup described here a thicker membrane of 200 nm was used, the irradiation dose can be calculated based on this value (see supplementary, Table S1). Moreover, simulations of electron scattering and of plasmid diffusion suggest that the kinetic energy spectrum of the electrons throughout the water is dominated by low energy electrons. The energy of these electrons is below 100 eV and on average in the range of 50 eV^27^. These data allow to estimate the kinetic energy of electrons in the vicinity of DNA. Thus, the electrons used exhibit energies far above the ionization thresholds of water and DNA, which are approximately 7 to 8 eV^39–42^ for DNA and around 10 eV for water.

## Conclusion

DNA double-strand breaks are the most critical radiation-induced damage, which eventually will lead to cell death. Most ionizing radiation in water ends in an avalanche of low energy electrons, which are together with hydroxyl radicals the key players in damaging DNA. To get closer to the physiological conditions in living cells, a special sample holder had been developed^12^ that allowed electron irradiation of DNA in aqueous solution and helped to evaluate the potential of ectoine in protecting DNA.

In water without ectoine, the applied electron radiation caused multiple double-strand breaks in plasmid pUC19 as witnessed by the generation of highly fragmented DNA. In contrast, 1 M ectoine preserved intact the native supercoiled conformation of plasmid DNA and suppressed any damage even at a high irradiation dose of 23.8∙10^12^ primary electrons with 30 keV, which corresponds to an effective dose of 15.8 Gy.

Until now, the mechanism on how ectoine protects the stability of plasmid DNA is not fully understood. The replacement of the (first) hydration shell of DNA by ectoine could possibly lead to this effect, because less secondary electrons will be generated by interaction of ionizing radiation with water in the vicinity of the DNA. It is, however, also possible that ectoine acts as a radioprotector in terms of its electron as well as radical scavenging properties.

Therefore, further studies have to clarify the precise mechanism by which ectoine protects DNA. These studies have to include stability tests for ectoine for electron and UV^43^ exposure to find out if ectoine undergoes irreversible chemical changes. We already know from our recent work that ectoine protects DNA from radiation in a concentration-dependent manner^44^. Furthermore, ectoine must be compared in terms of radiation protection to other compatible solutes such as hydroxyectoine, trehalose and glycine betaine. Comparing the different solutes will help to determine whether the specific tetrahydropyrimidine structure of ectoine contribute in stabilizing DNA or whether more common features contribute to the protection, for instance the relatively large hydrophobic surface area of compatible solutes combined with good water solubility. Experiments with known OH-scavengers such as DMSO must be included as well. Moreover, our experiments, which we have carried out in a simple model system have to be expanded to chromosomal DNA and even whole cells. Interestingly, ectoine is not taken up by a number of eukaryotic cells (skin cells) but it is not clear yet whether this is true for all eukaryotic cells (e.g. kidney cells).

In conclusion, this work provides strong evidence in support of ectoine as a potent and specific substance in protecting DNA against ionizing radiation. If taken up by eukaryotic cells, ectoine could be used as a radioprotector for DNA and possibly for cells in a wide-ranging of medical applications such as cancer radiation therapy.

## Experimental Procedures

### Irradiation of plasmid DNA

The plasmid created at the University of California (pUC19, 2686 base pairs) was purchased at New England Biolabs GmbH. The plasmid pUC19 was desalted by Microcon YM-50 filters and resuspended in ultrapure water (conductance 0.055 µS cm^−1^, pH 6.6) The concentration and purity of plasmids was determined by using NanoDrop2000c (Thermo Scientific). The plasmid sample at concentrations of 50 ng/μl were adjusted in ultra-pure water (conductance 0.055 µS cm^−1^, pH 6.6) or ectoine (1,4,5,6-tetrahydro-2-methyl-4- pyrimidinecarboxylic acid, purchased from bitop AG, Witten, Germany) solution (1 M, pH 6.6). A volume of 4 μl pUC19 solution were used for irradiation. As described by Hahn *et al*.^12^ the irradiation of plasmid DNA were performed through a vacuum-separating nanomembrane in aqueous solution with a FEI XL30 scanning electron microscope (SEM) by use of primary electrons with kinetic energies of 30 keV. The irradiation was performed at room temperature. Currents of 0–12.7 nA (determined with a faraday cup and a Keithley 6485 picoammeter) with irradiation times of (300 ± 4)s were used. The SE-detector voltage was fixed at 0 V to enable calculations of the dosis. The sample pre-preparation, the electron irradiation and post-preparation of the samples were carried out exactly within 2 h. After sample treatment, the plasmid samples were stored at −20 °C (freezing and thawing only 1 time) before analyzing by using gel electrophoresis and atomic force microscopy.

### Agarose gel electrophoresis

Gel electrophoresis was performed to identify different plasmid structures and to determine topological changes after electron irradiation of plasmids. Each channel of 1% agarose gel was run with 75 ng plasmids at 50 V for 90 minutes in gel electrophoresis. The DNA was stained with GelRed^I^ (GeneON GmbH) and visualized as well as quantified by measuring the fluorescence intensity of each plasmid structure (Herolab E.A.S.Y® Doc plus, E.A.S.Y Win-Software).

### AFM sample preparation

Atomic force microscopy was performed to determine the structure of DNA plasmids in ectoine-aqueous solution after electron irradiation. The samples were prepared on mica, which is commonly used as ultra-smooth substrate for the deposition of biomolecules. The surface was functionalized based on an approved procedure^45^ by incubating an aliquot of 20 µl of 0.05% APTES solution ((3-Aminopropyl)-triethoxysilane) for one minute at room temperature on freshly cleaved mica and by purging with ultrapure water (conductance 0.055 µS cm^−1^). Nitrogen (Linde, 5.0) was used to blow-dry the functionalized mica plates. Under these conditions an APTES layer with a thickness < 1 nm was achieved as checked with AFM after scratching. An aliquot of plasmid DNA (10 µl with 2 ng/µl in 1 mMol Tris-HCl buffer, pH 7.5) was deposited on the APTES treated mica, incubated for two minutes and purged at least three times with ultrapure water to remove unbound molecules. The samples were blow-dried and stored in a glass vessel with phosphorus pentoxide to reduce humidity RH to <15%.

### Intermittent contact atomic force microscopy

Intermittent contact AFM was performed in air by using a Nanotec Electronica SL (Madrid, Spain) microscope. In order to provide high spatial resolution cantilevers equipped with diamond-like carbon (DLC) whiskers at the tip end (supplier: NT-MDT) were used, which have a curvature radius of about 1–3 nm according to the manufacturer. Two different types of cantilevers were used (126 ± 10 kHz for NSG01_DLC, 295 ± 10 kHz for NSG10_DLC) without apparent influence on the quality of images. In all cases it was checked if conventional feedback on amplitude of vibration or PLL-based feedback using different frequencies yields best result. Images were collected at a scan frequencies between 1–2 Hz and the fixed scan size was chosen to 2.5 µm since statistical purposes require a large number of plasmids on each AFM image. Thus a lateral pixel resolution of ~4.9 nm is achieved. Extreme care was required to adjust the feedback in a way that nor the DNA is destroyed neither the tip is contaminated. Relative humidity in the closed chamber was reduced by a desiccant, however sample surfaces will still exhibit adsorbed and absorbed water, influencing tip-sample adhesion and energy dissipation. Thus, topography images appear not as sharp as images of the amplitude or phase signal which consistently were used for analysis. Since only conformation as well as contour length of the plasmids is analyzed, the information of the DNA height is not required.

### AFM image post-processing

For visualization, analysis and post-processing of the AFM images WsXM software (version 4.0 Beta 6.4)^46^ was used. Since the immobilization procedure causes artefacts like silan aggregates bright spots in the images were suppressed. If necessary, a post-processing in terms of a low-pass-filter was applied after the carefully performed background subtraction of artefacts caused by substrate or scanning process. In all cases the color scale was optimized to enhance the plasmid contour.

### Data analysis and quantification

Analyzing gel electrophoresis data, the amount of undamaged plasmids of electron irradiated samples were quantified, with (1 M) and without ectoine in the aqueous solution. Additionally, within the irradiated samples four different characteristic representatives (without ectoine, 0.2 Gy and 14.07 Gy respectively/ectoine solution, 0.26 Gy and 15.83 Gy respectively) were selected to compare the fraction of supercoiled, open circular and linearized plasmids with the results from AFM experiments. In AFM experiments three replicates per condition were deposited on mica and at least three different parts of each sample were imaged. For each experimental condition, not less than 11 AFM images on 2–3 mica samples were captured. By doing so, typically several hundreds DNA plasmids were included in the analysis for each sample. A visual assessment of each image was carried out by two independent persons in order to count the amounts of different plasmid structures. The following criteria were used: 1) Plasmid structures have to be completely visible on the image. 2) Plasmids have to be recognizable as single molecule and not lie on top of each other. 3) The classes of different plasmid isoforms are: supercoiled, open circular or linear structure. 4) The contour length of the linear structure (lin DNA) was found to be approximately 913 nm as expected. Since silanization of mica formed a granular substructure the length of strongly fragmented linear DNA with less 100 bp (~34 nm) could not be counted properly. For statistical analysis of AFM data each experimental condition was compared due to the mentioned criteria and the topological differences in plasmids. Here, the procentual amounts of each plasmid structure were taken as a basis, not the actually counted from every image. Results are expressed as the mean for each fraction.

The datasets generated during and/or analysed during the current study are available from the corresponding author on reasonable request.

## Electronic supplementary material

Supplementary Table S1
