# Supplementary material for: Ectoine protects DNA from damage by ionizing radiation
Source: Sci Rep. 2017 Nov 10;7:15272. doi: 10.1038/s41598-017-15512-4 (PMC5681641; doi:10.1038/s41598-017-15512-4)
Supplement: Supplementary file 1 — Supplementary Table S1 [file 41598_2017_15512_MOESM1_ESM.pdf]

Supplementary information

Ectoine protects DNA from damage by ionizing radiation

M.- A. Schröter[1], S. Meyer[1,2], M. B. Hahn[1,3], T. Solomun[1], H. Sturm[1,4], H. J. Kunte[1*]

[1]Federal Institute for Materials Research and Testing, D-12205 Berlin, Germany
[2]Institute of Biochemistry and Biology, University of Potsdam, D-14476 Potsdam, Germany
[3]Institute of Experimental Physics, Free University Berlin, Department of Physics, D-14195 Berlin, Germany.
[4]Technical University Berlin, D-10587 Berlin, Germany

S.M. and M.-A.S. contributed equally to this work and should be considered as co-first authors.

*Hans-Joerg.Kunte@bam.de

Supplementary Table S1| Irradiation of pUC19 in water and in 1 M ectoine solution. Acceleration voltage: 30 kV, irradiation time: 300±4 s. Currents, number of primary electrons are measured, the calculation of irradiation doses is based on Hahn *et. al.*[28] (see reference in the main text.) Total error of the currents is less 5%. All samples are analyzed by gel electrophoresis. Samples, which are additionally analyzed with atomic force microscopy, are bold written.

**pUC19 in water**	**current [nA]**	**number of primary electrons [$\cdot 10^{12}$]**	**effective irradiation dose [Gy]**	**pUC19 in 1M ectoine solution**	**current [nA]**	**number of primary electrons [$\cdot 10^{12}$]**	**effective irradiation dose [Gy]**
control 1	*0*	*0*	*0*	*control 1*	*0*	*0*	*0*
control 2	*0*	*0*	*0*	*control 2*	*0*	*0*	*0*
sample 1	0.107	0.200	*0.13*	sample 1	0.115	0.216	0.14
**sample 2**	**0.163**	**0.305**	***0.20***	**sample 2**	**0.210**	**0.394**	**0.26**
sample 3	0.285	0.534	0.36	sample 3	0.290	0.544	0.36
sample 4	0.365	0.684	0.46	sample 4	0.448	0.84	0.56
sample 5	0.416	0.780	0.52	sample 5	0.542	1.02	0.68
sample 6	0.419	0.786	0.52	sample 6	0.551	1.033	0.69
sample 7	0.503	0.943	0.63	sample 7	0.551	1.033	0.69
sample 8	0.612	1.15	0.76	sample 8	7.52	14.1	9.39
sample 9	0.750	1.41	0.94	sample 9	7.54	14.14	9.41
sample 10	0.849	1.59	1.06	sample 10	8.05	15.09	10.05
sample 11	1.02	1.91	1.27	sample 11	9.27	17.38	11.57
sample 12	3.10	5.81	3.87	sample 12	9.37	17.57	11.7

sample 13	4.47	8.38	5.58	sample 13	11.21	21.02	13.99
sample 14	6.22	11.67	7.76	sample 14	11.23	21.06	14.02
sample 15	7.35	13.78	9.17	sample 15	12.50	23.44	15.6
sample 16	10.65	19.97	13.29	sample 16	12.52	23.48	15.63
**sample 17**	**11.28**	**21.14**	**14.07**	**sample 17**	**12.68**	**23.78**	**15.83**
